# Leptin plays a role in the multiplication of and inflammation in ovarian granulosa cells in polycystic ovary syndrome through the JAK1/STAT3 pathway

**DOI:** 10.1016/j.clinsp.2023.100265

**Published:** 2023-08-08

**Authors:** Xuan Zhao, Yaming Xiong, Ya Shen

**Affiliations:** a Obstetric and Gynecologic Ultrasound Department, Wuhan Children's Hospital (Wuhan Maternal and Child Healthcare Hospital), Tongji Medical College, Huazhong University of Science & Technology, Wuhan, China; b Ultrasound Department, JingMen No.1 People's Hospital, Jingmen, China; c Department of Gynaecology and Obstetrics, Wuhan Children's Hospital (Wuhan Maternal and Child Healthcare Hospital), Tongji Medical College, Huazhong University of Science & Technology, Wuhan, China

**Keywords:** PCOS, Granulosa Cell, LEP, JAK1, STAT3, Apoptosis, Inflammation

## Abstract

**Objectives:**

The authors determined the level of Expression of Leptin (LEP) in Polycystic Ovary Syndrome (PCOS) patients with or without obesity and in GCs treated with insulin.

**Methods:**

LEP expression was first assessed in ovary cortex specimens collected from women with PCOS with or without obesity as well as from healthy controls. Ovarian Granulosa Cells (OGCs) induced by insulin extracted from a mouse model were used in further functional research.

**Results:**

Real-time PCR and western blotting indicated that LEP expression was upregulated in GCs induced by insulin, in comparison with that in GCs not induced by insulin. Furthermore, the knockdown of LEP resulted in a reduction in growth and multiplication and an increase in apoptosis and inflammation in GCs induced by insulin. Next, the authors evaluated the effect of LEP on three key pathways of inflammation (MAPK, NF-kB, and JAK1/STAT3); results showed that the JAK1/STAT3 pathway was induced by LEP knockdown, as evidenced by the upregulation of phosphor-JAK1, phosphor-STAT3, and nuclear STAT3 expression. Administration of curcumin, a specific inhibitor of STAT3, counteracted the effect of LEP knockdown on cell inflammation and apoptosis.

**Conclusion:**

The present data suggest that upregulation of LEP expression in the PCOS granulosa cell model is essential for reducing apoptosis and inflammation by modulating the JAK1/STAT3 pathway axis.

## Highlights

• LEP may be associated with the development of PCOS.
• LEP may be a protective factor against apoptosis in GCs treated with insulin.
• LEP knockdown induced JAK1/STAT3 activation could be reversed by administration of curcumin.

## Introduction

PCOS is a multifactorial disorder and affects approximately 5%‒10% of all fertile females [1]. PCOS is characterized by chronic anovulation, hyperandrogenemia, and polycystic ovaries, observed upon ultrasound scanning [2]. PCOS is one cause of Anovulatory Sterility (AS); nevertheless, the precise pathogenesis of PCOS remains unclear, although data highlight environmental considerations as well as a hereditary basis [3].

Leptin (LEP), belonging to the “tumor necrosis factor” family under cell factors, is an amino peptide composed of 167 amino chemichals [4]. LEP, secreted by adipose cells in fat tissues, moves in a free form or combined with a soluble isomer. In addition, levels of LEP are positively correlated with the number of fat cells [5]. In humans, the primary function of LEP seems to be the regulation of orexis and heat generation for weight control. High leptin levels have been confirmed to stimulate the appestat, which can lead to reduced heat generation [6]. It exerts interferential effects on the maturity of female egg cells as well as activation of ovarian enzymes that participate in steroid generation (e.g., Follicle-Stimulating Hormone [FSH] and Luteinizing Hormone [LH]) [7]. LEP has also been suggested to be a sign of periphery, showing whether the nutriture of reproductive functions is adequate [8]. However, studies with conflicting results have been obtained regarding the regulation of LEP by sex steroid/pituitary gonadotropic hormones.

LEP has been demonstrated to have a positive effect on proinflammatory responses. It can stimulate the release of chemotactic agents and Reactive Oxygen Species (ROS) in neutrophils [9]. A previous study suggested that LEP expression is positively correlated with the number of apoptotic cells in KGN cell lines [10]. Nevertheless, studies have not investigated the role and underlying mechanisms of action of LEP in PCOS. In addition, the potential mechanisms underlying inflammation and apoptosis have been published. In this study, the authors aimed to determine the role and mechanism of action of LEP in PCOS, especially its association between inflammation and apoptosis. The authors determined the level of expression of LEP in PCOS patients with or without obesity and in GCs treated with insulin. Dysregulation of LEP expression resulted in robust inflammation, elevated apoptosis, and reduced viability of GCs. The association between LEP and JAK1/STAT3 in GCs treated with insulin has been investigated.

## Material & methods

### Clinical specimens

PCOS subjects (*n* = 20) at Wuhan Children's Hospital (Wuhan Maternal and Child Healthcare Hospital), Tongji Medical College, and Huazhong University of Science & Technology were screened. This study was approved by the local institutional ethical review board of Wuhan Children's Hospital (Wuhan Maternal and Child Healthcare Hospital), Tongji Medical College, Huazhong University of Science & Technology (protocol number 2022R052-E01). The clinical features of these patients showed in Table 1. All participants provided informed consent before being included in this investigation. Twenty PCOS subjects agreed for the assessment of sterility via laparoscopy. PCOS was diagnosed according to the guidelines of the Rotterdam European Society of Human Reproduction and Embryology (ESHRE) with revisions (2003) [11]. Ten women with eumenorrhea, those accepting sterilization, and those with benign mastectomy, as revealed via laparoscopy, were included in the control group. All subjects underwent health examinations (including hipline, weight, waistline, and height measurements). Additionally, an improved Ferriman-Gallwey score (mFG) for polytrichosis was assigned. The authors measured their body mass index and evaluated their ovaries. The authors also assessed fasting insulin levels. The indicated evaluations were subsequently conducted for 12 hours of fasting throughout the night. In PCOS subjects, levels of fasting insulin, TT, homeostatic model assessment of insulin resistance, and mFG were increased compared with those in the control group.

**Table 1:** Clinical characteristics of patients in control and PCOS groups.

Characteristics	Control (*n* = 10)	PCOS (*n* = 20)	*p*-value
Age (years)	26.3 ± 3.1	27.5 ± 4.0	0.061
Body mass index (kg/m^2^)	20.1 ±1.7	20.3 ± 2.0	0.200
Follicle stimulating hormone (IU/L)	7.6 ± 0.7	4.1 ± 0.6	0.001
Luteinizing hormone (IU/L)	22.8 ± 1.8	42.3 ± 2.7	0.009
Prolactin (mg/mL)	10.1 ± 0.9	15.8 ± 1.3	0.001
Estradiol (pg/mL)	30.9 ± 2.2	57.8 ± 5.1	0.002
Glucose (mmoL/L)	6.3 ± 0.7	3.1 ± 0.3	0.001
Testosterone (nmoL/L)	2.4 ± 0.3	5.2 ± 0.5	0.001
Hyperandrogenism % (n)	1 (10.0)	8 (40.0)	0.004
Normal androgen% (n)	7 (70.0)	7 (35.0)	0.008
Insulin (U/L)	5.9 ± 0.6	17.0 ± 1.9	0.001
Hyperinsulinemia % (n)	1 (10.0)	7 (35.0)	0.022
Number of mature oocytes	17.9 ± 1.5	7.2 ± 1.3	0.001
Number of good quality embryos	10.1 ± 1.2	5.1 ± 0.7	0.001

### In vitro GC culturing

Female-CF1™ mice (19 days old) received intra-peritoneal injections of 5 units of eGC (FSH analog; Calbiochem, San Diego, CA) to trigger follicle production. Afterward, the mice were administered 5 units of hCG (LH analog; Sigma, St. Louis, MO) 48 hours following eCG administration to trigger luteinization and ovulation. Ovaries were removed 12 hours after therapy. A follicle puncture with phosphate-buffered saline was conducted to acquire GCs from the ovary specimens. Fibronectin-coating plates with 6 wells for culturing GCs (2.5 × 10^4^ cells per well) were used for assessing the expression levels and cell death. Proteins were evaluated in tissue dishes (1.5 × 10^6^ cells/10 cm). The segregated GCs were treated with Trizol (Invitrogen, Carlsbad, CA) before culturing to examine the expression level “before culture”. DMEM/F12 containing 10% FBS and 1% GEN was selected for culturing the cells under 5% CO_2_ and 37°C. Six-well plates were selected for seeding the cells; the cells were treated with 100 mg/mL insulin and cultured for 24 hours, for the following experiments.

### Cell transfection

The shRNA-Negative Control (NC) and shRNA-LEP were designed and synthesized by RiboBio (Guangzhou, China). The cells were cultured in 6-well plates (density: 5 × 10^5^/well) until they reached a confluence of 80%, followed by transfection with control/overexpression vectors using Lipofectamine™ 2000 (Invitrogen, Carlsbad, CA, USA). The transfection efficiency of GCs was approximately 60%. After transfection, the cellular activity was sustained for 96 hours.

### RNA separation and real-time PCR

Trizol reagent was selected for isolating RNA from the cells following treatment. The isolated RNA was quantified using SYBR® Green with a Roche LightCycler® 480 RT-PCR system (Roche, Germany). Glyceraldehyde-3-phosphate dehydrogenase was used as an internal control. Subsequently, RT-PCR was conducted with SYBR™ Green PCR Master Mix (final volume: 20 µL). Evaluated targeting values according to the 2^−delta delta CT^ method with conversion to internal controls, in which the method for controlling specimens is selected as the calibrator.

### Western blot analysis (WB)

RIPA buffer containing a protease inhibitor cocktail (Roche Applied Science) was used to trigger cellular disruption. The BCA Protein Assay Kit was selected for quantifying proteins, which were extracted via 10% sodium dodecyl sulfate-polyacrylamide gel electrophoresis and subsequently transferred to 0.45-micron polyvinylidene fluoride membranes (Millipore, MA, USA). The membranes were blocked with 5% bovine serum albumin for 1h at 25°C. Next, the membranes were incubated with specific antibodies overnight at 4°C, followed by washing with Tris-buffered saline with Tween 20. Next, the cells were incubated with secondary antibodies at 25°C for 1h. Immune reactivity was assessed with a SuperSignal™ West Femto Maximum Sensitivity Substrate Kit (Thermo) with a C-DiGit® Blot Scanner.

### MTT assay

Cell growth was evaluated using an MTT assay. In brief, cells were treated with 20 µL MTT (0.5 mg/mL). Next, the supernatant was discarded and 150 µL of DMSO was added to all wells. Plates were then rotated for 10 minutes to dissolve the formazan dye. Absorbance at a wavelength of 490 nm was measured using an Infinite M200 microplate reader (Tecan, Männedorf, Switzerland).

### Cell counting kit-8 (CCK-8) assay

Cellular viability was detected using the CCK-8 assay. Initially, the authors seeded the cells onto 96-well plates. The CCK-8 reagent (0.01 mL) was added to all wells at various time points after transfection. The plates were cultured for 2h at 37°C. Next, absorbance was measured at 450 nm using an Infinite® M200 plate reader (Tecan, Männedorf, Switzerland).

### Colony formation

For the colony formation test, cells were placed in 6-well plates for 1 week. Subsequently, the cells were fixed in 4% methyl alcohol for 20 min and then stained with 1% crystal violet.

### Enzyme-linked immunosorbent assay (ELISA)

The supernatant from the cell cultures was obtained to detect secreted interleukin-1β (IL-1β), interleukin-6 (IL-6), interleukin-18 (IL-18), and tumor necrosis factor-alpha (TNF-α) using ELISA kits (R&D Systems, Minneapolis, MN, USA). Absorbance at 450 nm was detected using a Bio-Rad Laboratories Model 680 microplate reader (Bio-Rad, Hercules, CA, USA). The concentrations of all proteins were then determined from the standard curve according to a previously described method [12].

### Cell apoptosis determination

Annexin V-FITC/propidium iodide flow cytometric assay kits were used to assess cell death. In brief, 20 µL of binding buffer was added for cellular re-suspension, followed by subsequent incubation with 5 µL of PI and 10 µL of Annexin V-FITC for 20 min in the dark. Cell death was evaluated using Flow Cytometry (FC).

### Statistical analysis

The obtained data are expressed as the mean ± SD. A two-tailed *t*-test and one-way ANOVA were conducted to assess differences within multiple and two groups, respectively. Statistical significance was set at *p* < 0.05.

## Results

### LEP expression was promoted in the ovaries of PCOS patients with obesity and GCs treated with insulin

**Fig. 1:**
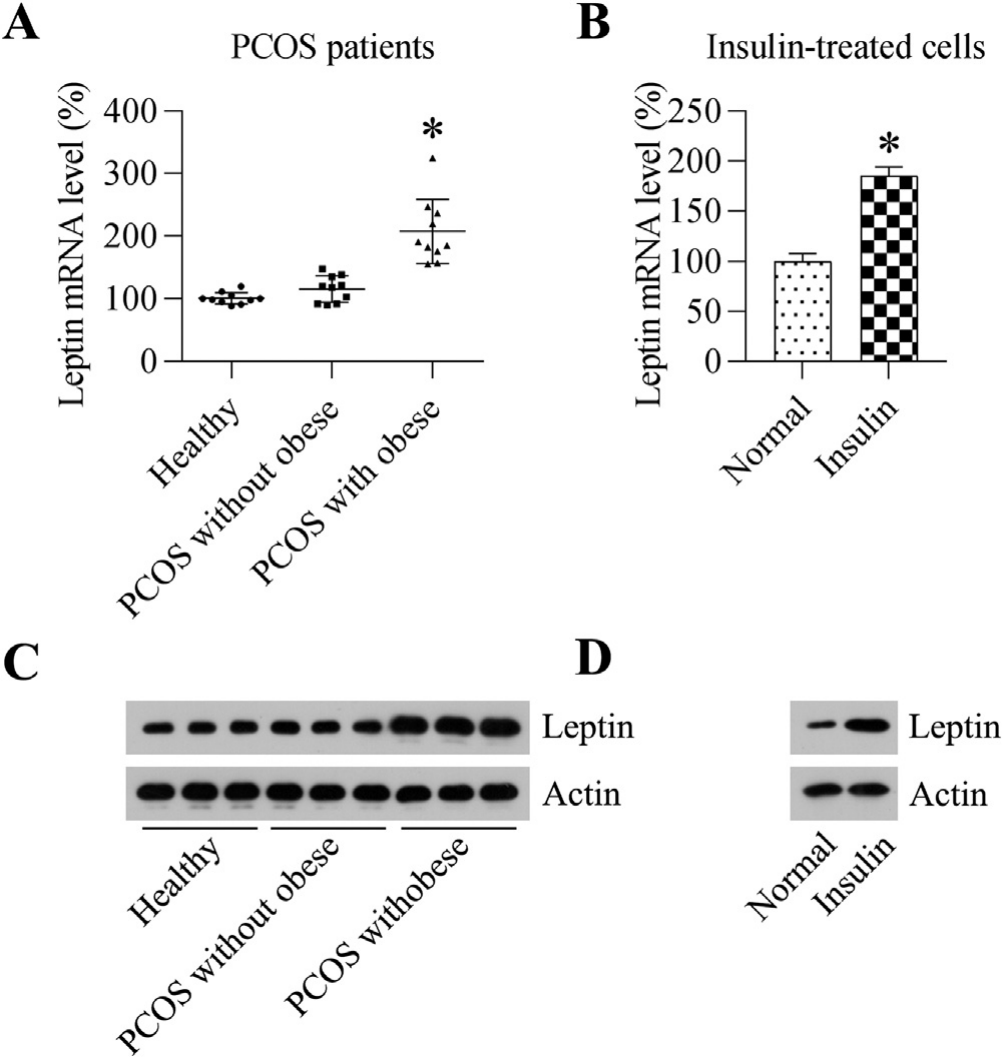
Expression of LEP in PCOS patients and GCs treated with insulin. (A) Real-time PCR analysis was used to detect the mRNA expression level of LEP in PCOS patients with obesity (*n* = 10), PCOS patients without obesity (*n* = 10) and healthy controls (*n* = 10). (B) qPCR analysis was conducted for detecting the mRNA expression level of LEP in GCs subjected to insulin treatment or no insulin treatment. (C) WB analysis was used to detect the protein expression level of LEP in PCOS patients with obesity (*n* = 10), PCOS patients without obesity (*n* = 10) and healthy controls (*n* = 10). (D) WB analysis was conducted for detecting the protein expression level of LEP in GCs subjected to insulin treatment or no insulin treatment. The data obtained from three independent tests are expressed in the form of mean ± SD (*p < 0.05).

To understand the role of LEP in PCOS, the authors first tested its expression level in the ovaries of PCOS patients with obesity (*n* = 10) or without obesity (*n* = 10) as well as in ovary tissues from the Normal Control group (NC). RT-PCR and WB assays showed that LEP levels were not altered in the ovaries of PCOS patients without obesity and in females from the control group (Fig. 1A and 1C). Furthermore, LEP expression in obese PCOS patients was remarkably upregulated compared to that in NCs and PCOS patients without obesity (Fig. 1A and 1C). Moreover, because hyperinsulinemia is one of the major features of PCOS, the authors selected treated GCs with insulin to construct a PCOS model. RT-PCR and WB suggested that LEP expression was induced by insulin stimulation (Fig. 1B and 1D). These data suggest that LEP may be associated with the development of PCOS.

### Role of LEP in the multiplication and apoptosis of and inflammation in GCs treated with insulin

**Fig. 2:**
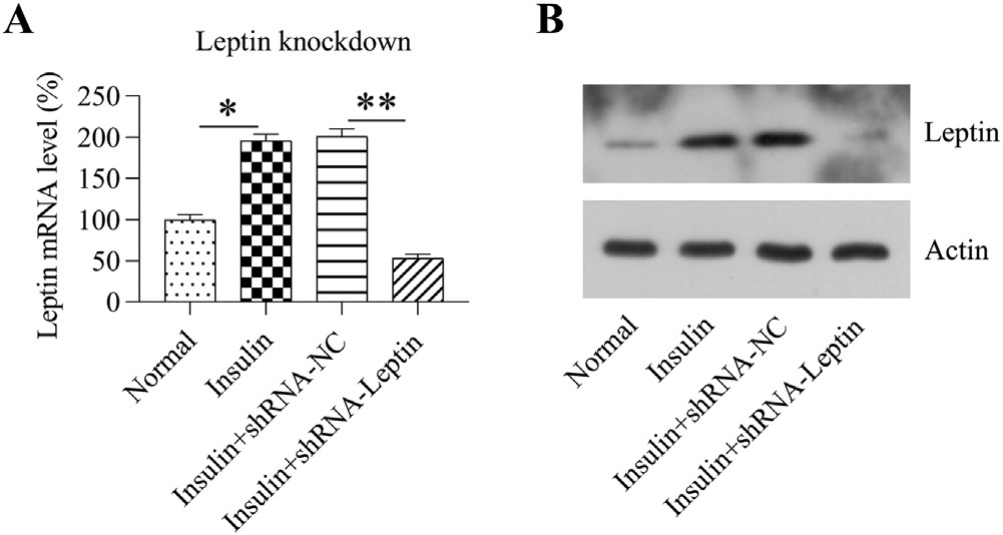
Silencing of LEP in GCs treated with insulin. GCs were treated with a high concentration (100 mg/mL) of insulin and then subjected to transfection with shRNA-NC or shRNA-LEP for 24h. (A‒B) qPCR and WB analyses were conducted to detect the mRNA and protein levels of LEP in granulosa cells, respectively (*p < 0.05, ** *p* < 0.01).

As an association between LEP and inflammation and apoptosis has been indicated previously [9,10], the authors examined the effects of LEP on inflammation and cell death in GCs treated with insulin. First, GCs treated with insulin were subjected to transfection using shRNA-LEP for 24h. RT-PCR and WB confirmed that LEP expression was significantly decreased following transfection with shRNA-LEP (Fig. 2A and 2B).

**Fig. 3:**
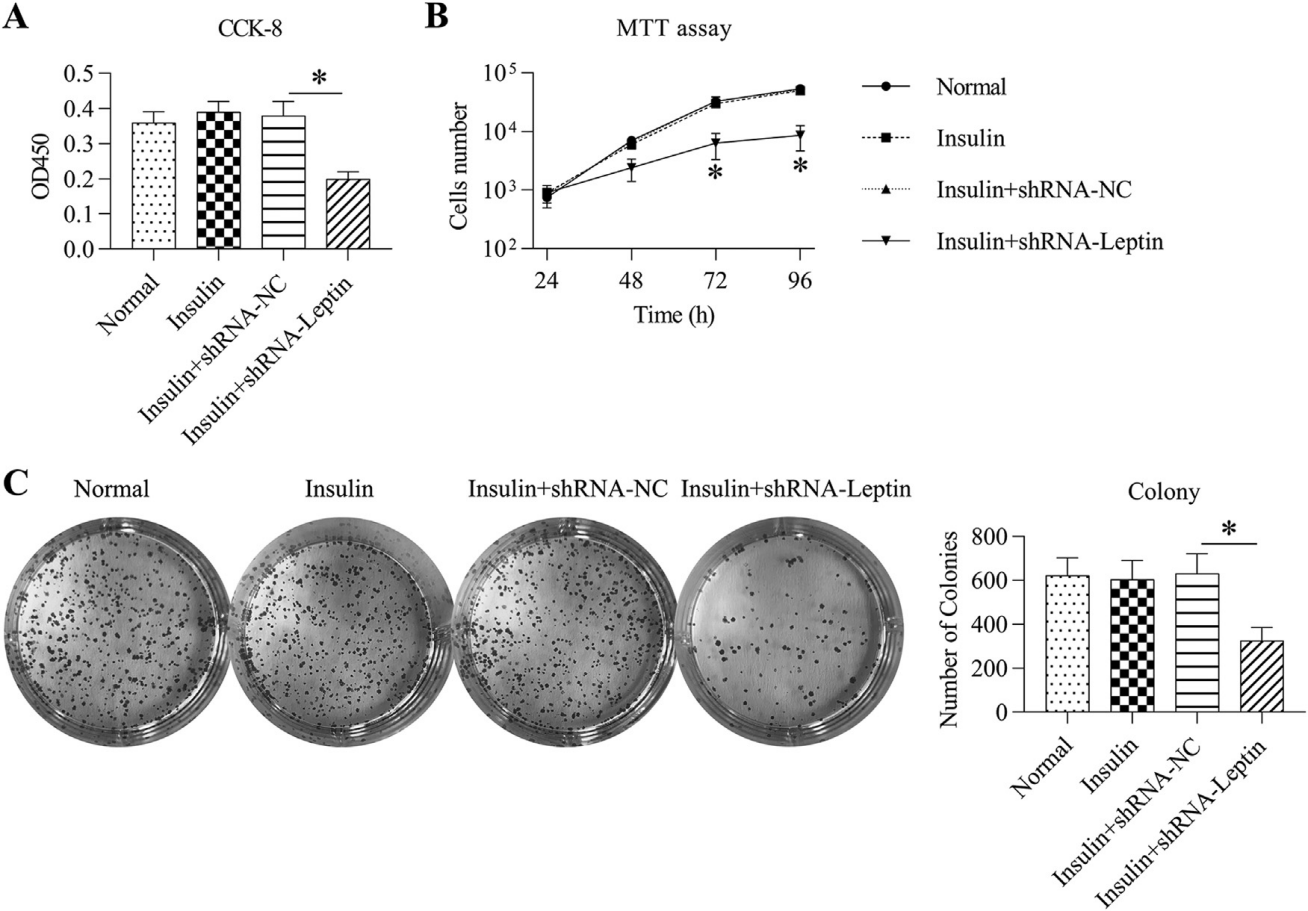
Influence of LEP silencing on the viability of GCs treated with insulin. (A) Cell counting kit-8 assay was used for detecting the activity of GCs treated with insulin. (B) MTT assay was employed for determining the multiplication of insulin-treated GCs subjected to transfection with shRNAs. (C) Colony formation of the granulosa cell lines subjected to transfection with shRNAs (*p < 0.05 vs. Insulin group * *p* < 0.05).

Cell viability, proliferation, and growth were assessed using CCK-8, MTT, and colony formation assays. Compared with the insulin+shRNA-NC group, the LEP-knockdown group showed significantly reduced cell viability (Fig. 3A). Furthermore, the MTT test was utilized for determining GCs treated with insulin. Transfection with shRNA-LEP caused an obvious reduction in cell number at 24‒96 hours post-transfection (Fig. 3B). In addition, colony formation assays revealed that the number of colonies generated from insulin-treated and LEP-depleted cells was significantly lower than that in insulin-treated control cells (Fig. 3C).

**Fig. 4:**
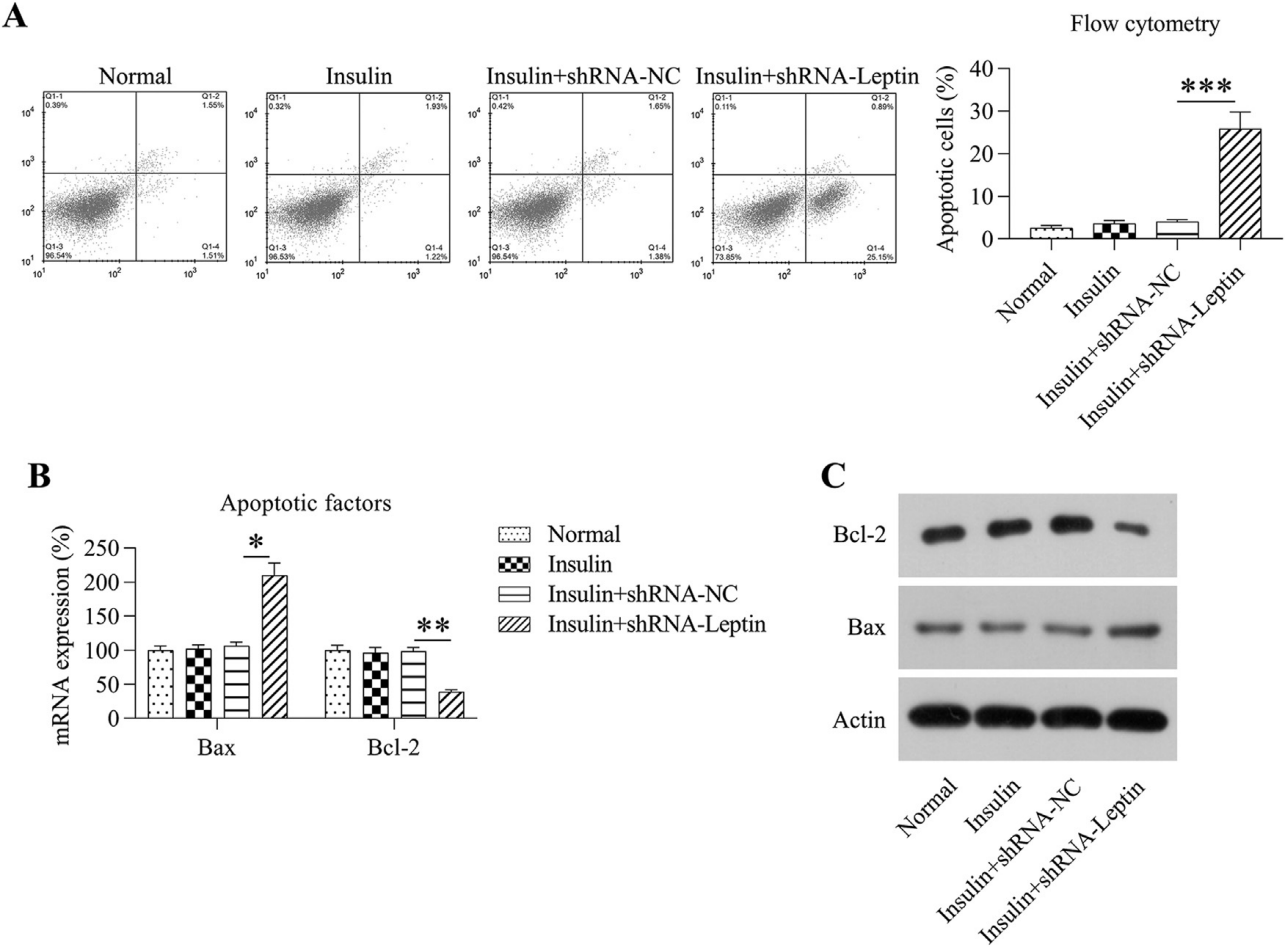
Influence of LEP silencing on death of GCs treated with insulin. (A) Annexin V-FITC/propidium iodide flow cytometric assay indicated the degree of cell death in GCs treated with insulin. The histogram indicates the level of death of GCs following multiple therapeutic methods. (B‒C) WB analysis was conducted for detecting the mRNA and protein expression levels of B-Cell Leukemia/Lymphoma 2 and Bax in GCs treated with insulin (*p < 0.05 vs. Insulin group. * *p* < 0.05, ** *p* < 0.01, *** *p* < 0.001).

Death of insulin-treated GCs showing downregulated LEP expression was measured via Annexin V-FITC/propidium iodide flow cytometric assays. The authors found a depletion of LEP upregulated GC cell death compared with that in the insulin+shRNA-NC group (Fig. 4A). Meanwhile, Bax expression was induced by LEP knockdown, whereas the Bcl-2 level was reduced, as assessed by RT-PCR and WB (Fig. 4B and 4C). These results show that LEP may be a protective factor against apoptosis in GCs treated with insulin.

**Fig. 5:**
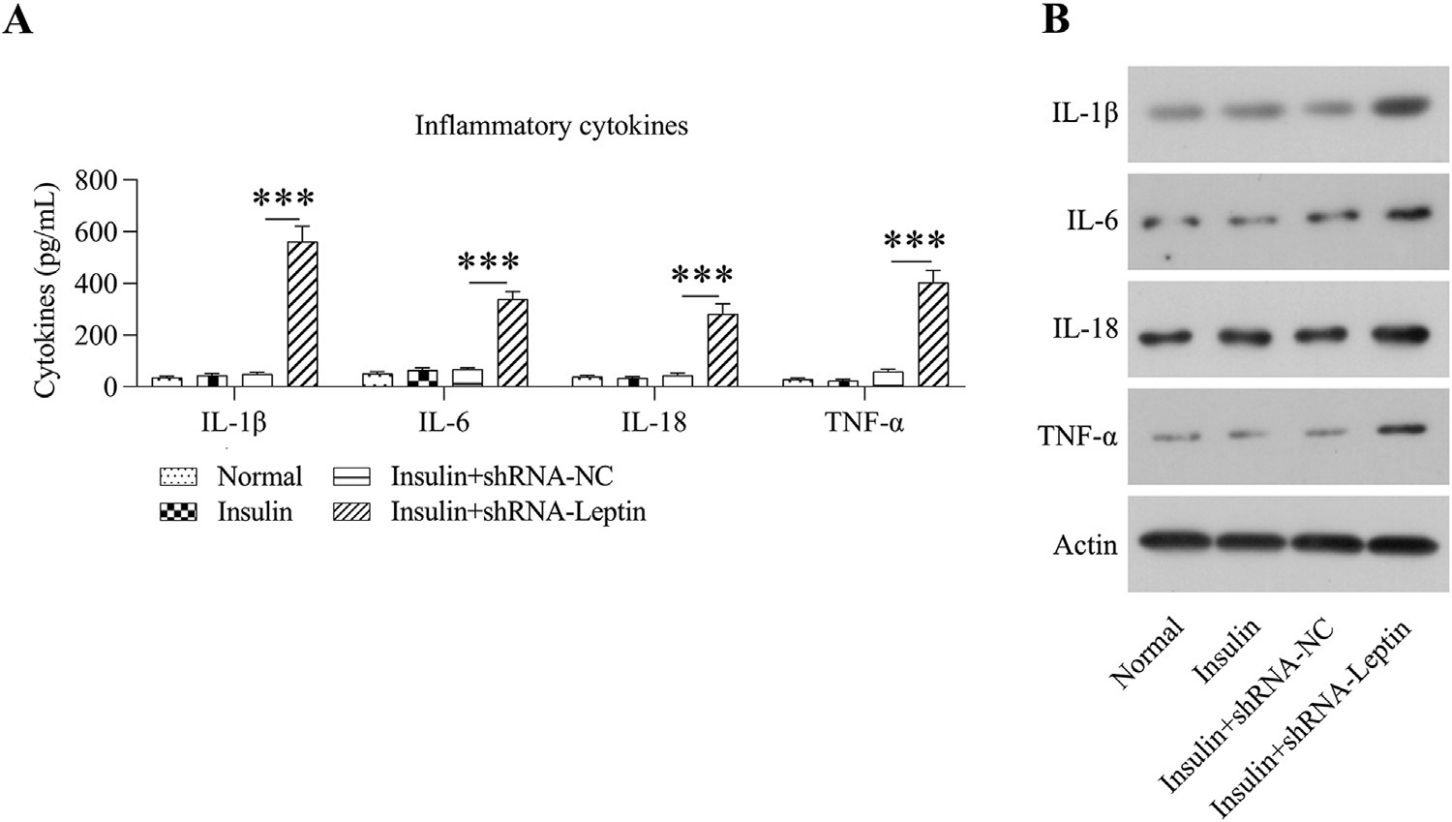
Influence of LEP silencing on inflammation in GCs treated with insulin. (A) ELISA was conducted for assessing the levels of interleukin-1β, interleukin-6, interleukin-18, and tumor necrosis factor-alpha in the culture supernatant of GCs treated with insulin. (B) WB was conducted for assessing the expression of interleukin-1β, interleukin-6, interleukin-18, and tumor necrosis factor-alpha in GCs treated with insulin (***p < 0.001).

Inflammation in GCs was detected by examination of IL-1β, IL-6, IL-18, and TNF-α using ELISA and WB. Release of IL-1β, IL-6, IL-18, and TNF-α was induced in GCs treated with insulin following LEP knockdown, as revealed via ELISA (Fig. 5A). WB also confirmed that the secretion of these four cytokines could be induced by LEP depletion (Fig. 5B).

### The JAK1/STAT3 pathway axis was activated in GCs processed with insulin by LEP knockdown

**Fig. 6:**
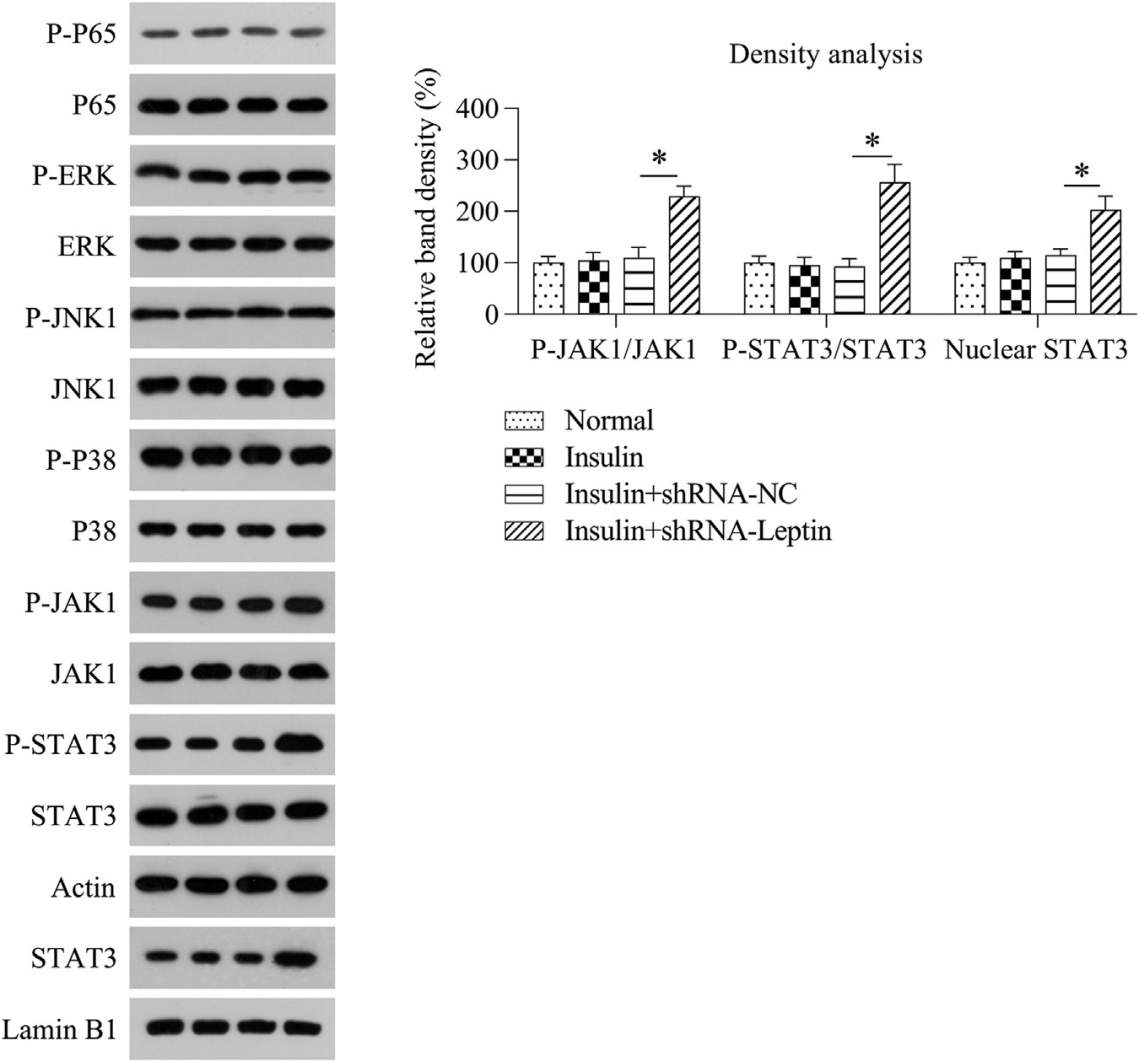
Effect of LEP silencing on activation of JAK1/STAT3 pathway in GCs treated with insulin. WB analysis displayed the expression level and phosphorylation of JAK1, STAT3, MAPKs, and NF-κB P65, as well as nuclear located STAT3 in the GCs treated with insulin via alternative transfection (*p < 0.05, ** *p* < 0.01), in comparison to the indicated group (*p < 0.05).

MAPK, NF-κB, and JAK1/STAT3 pathways are the three major signaling pathways contributing to inflammation; therefore, the authors determined the activation of these three pathways by examining phosphorylated JAK1, phosphorylated STAT3, nuclear STAT3, phosphorylated MAPK JNK1, phosphorylated MAPK P38, phosphorylated MAPK ERK, and phosphorylated NF-κB P65 levels. WB data showed that the MAPK and NF-κB pathways did not change after LEP knockdown. However, the JAK1/STAT3 pathway was activated due to LEP depletion in GCs treated with insulin, as evidenced by increased levels of phosphorylated JAK1, phosphorylated STAT3, and nuclear STAT3 (Fig. 6).

### STAT3 deactivation counteracted the effect of LEP on inflammation in and death of GCs treated with insulin

**Fig. 7:**
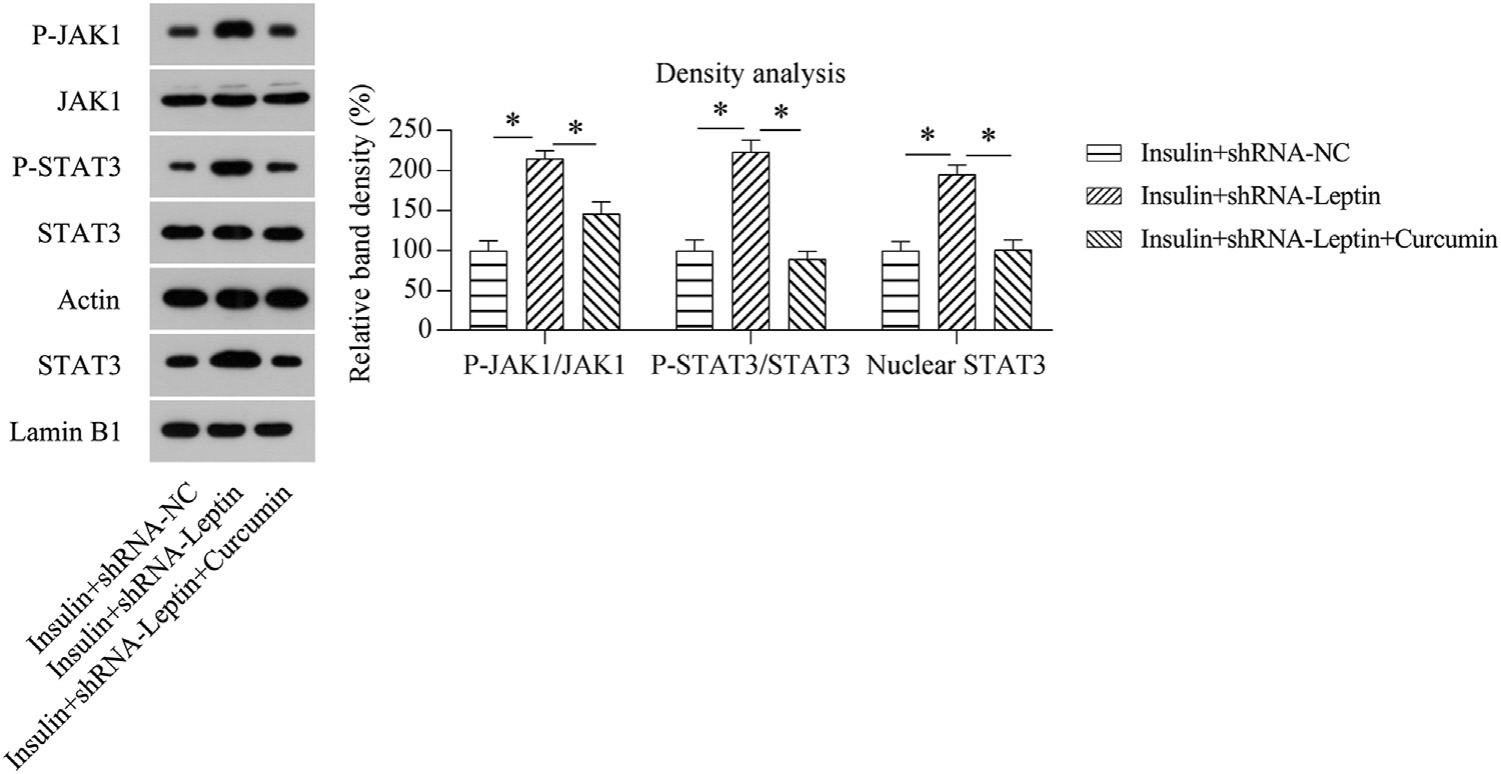
Administration of curcumin on activation of the JAK1/STAT3 pathway in GCs treated with insulin following LEP knockdown. GCs treated with insulin were subjected to transfection using shRNA-NC or shRNA-LEP for 12h, followed by treatment with 5 µM curcumin for 12h. WB analysis displayed the expression and phosphorylation of JAK1 and STAT3 as well as nuclear located STAT3 in GCs treated with insulin following different transfections (*p < 0.05).

Furthermore, the authors explored whether JAK1/STAT3 activation was attributed to the influence of LEP knockdown on GCs treated with insulin. Cells treated with insulin were subject to transfection with shRNA-LEP and/or treated with the specific STAT3 inhibitor curcumin. WB results confirmed that phosphorylated JAK1, STAT3 and nuclear STAT3 levels were significantly downregulated in cells treated with curcumin, in comparison with those in the group without treatment (Fig. 7), suggesting that LEP knockdown-induced JAK1/STAT3 activation could be reversed by administration of curcumin.

**Fig. 8:**
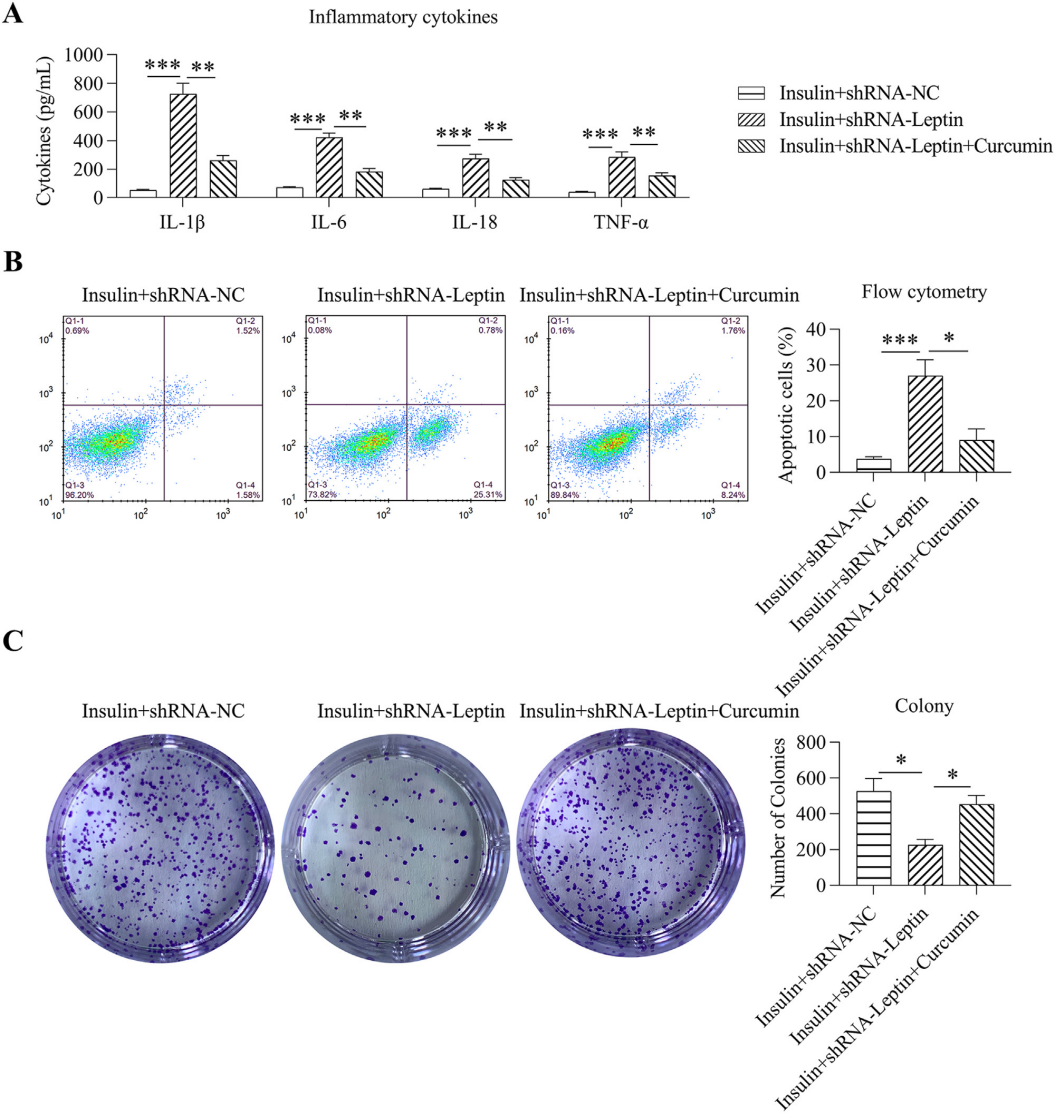
Effect of curcumin on inflammation, apoptosis, and colony formation of GCs treated with insulin following LEP knockdown. (A) ELISA was conducted for assessing the levels of interleukin-1β, interleukin-6, interleukin-18, and tumor necrosis factor-alpha in the culture supernatant of GCs treated with insulin. (B) Annexin V-FITC/propidium iodide flow cytometric assay indicated the degree of cell death in GCs treated with insulin. The histogram shows the level of death of GCs following multiple therapeutic methods. (C) Colony formation of the granulosa cell lines subjected to transfection with shRNAs (*p < 0.05, ** *p* < 0.01, *** *p* < 0.001).

To further evaluate the effects of curcumin administration on cell inflammation and apoptosis, an ELISA was performed in insulin-treated cells subjected to transfection with shRNA-LEP and treated with curcumin. Results showed that STAT3 deactivation significantly decreased the generation of proinflammatory cell factors in cells treated with insulin (Fig. 8A). FC indicated that STAT3 deactivation reduced the simulative functions of LEP knockdown on cell death (Fig. 8B). Furthermore, increased cell growth was observed in the cells treated with insulin and reduced following LEP and curcumin treatment, compared with that in non-treated cells (Fig. 8C). These data suggest that LEP knockdown-induced inflammation and death in GCs treated with insulin is STAT3 dependent.

## Discussion

PCOS is characterized by polycystic ovaries, hyperandrogenism, Insulin Resistance (IR), and persistent anovulation [13]. High LEP levels in women with PCOS seem to result from a positive relationship between serum LEP, Body Mass Index (BMI), and insulin [13] Among them, serum LEP was found to be significantly correlated with basal insulin levels, BMI, and insulin [14], suggesting the potential role of LEP in PCOS progression in obese patients. This investigation is the first attempt to elucidate the mechanism of action of LEP in cell activity, inflammation, and cell death in a PCOS model. A previous study indicated that STAT3, STAT1, and JAK1 could be found in GCs, especially in primitive follicles, showing that the JAK/STAT pathway is a conserved signaling pathway that interacts with GC functions in mammals [15]. However, the role of this important pathway in GC phenotypes has not been documented. In this study, LEP expression was found to be upregulated in PCOS patients with obesity and in GCs treated with insulin. LEP silencing impaired cell survival and elevated inflammation and cell death in insulin-treated GCs. Meanwhile, the JAK1/STAT3 pathway was found to be activated in GCs treated with insulin after LEP knockdown. Furthermore, the administration of curcumin in insulin-treated and LEP-downregulated GCs counteracted the effect of LEP knockdown on phenotypic changes in GCs. The present results showed that LEP is a modulatory molecule involved in this PCOS cell model and serves as an upstream modulator of JAK1/STAT3-associated inflammation and apoptosis in GCs treated with insulin.

Ovary tissues of PCOS patients are identified to present a state of chronic low-grade inflammation (involving high-grade leucocytes and generation of proinflammatory cellular factors) [16]. IL-6 is a pluripotent cell factor that mediates inflammatory reactions by controlling cellular differentiation, migratory ability, multiplication, and cellular death, thus influencing the progression of IR [17]. The generation of IL-6 is associated with ovarian androgen overproduction, which is one of the main pathological events in PCOS [18]. Other pro-inflammatory cell factors, including IL-1β, IL-18, and TNF-α, have been reported to participate in the potential mechanism underlying chronic phlogosis in PCOS [19,20]. A previous study demonstrated that LEP in chronic inflammation's osteogenesis and the increased LEP expression levels in adiposity seems to be conducive to a low-grade phlogosis background, which promotes weight gain and is associated with an increased likelihood of developing angiocardiography, type II diabetic disorders, and retrogressive disorders, including cancer and autoimmune disease 21, 22, 23. In this study, the authors observed that LEP depletion in GCs treated with insulin-induced a high level of inflammation, as evidenced by the excessive release of cytokines by ELISA and intracellular cytokines by WB. The authors further found that LEP depletion caused inflammation in a JAK/STAT-dependent manner, not in a MAPK and NF-κB dependent manner. This mechanism could be further confirmed by the alleviation of inflammation in insulin-treated and LEP-depleted GCs, caused by the administration of the specific STAT3 inhibitor curcumin.

The activation and deactivation of signal channels related to cell death have been reported as predominant factors in multiple disorders [24,25]. Apoptosis can equilibrate the vital capacity and death of neoplastic cells as a manifestation of programmed cell death. Dysregulation of cell death is related to poor clinical outcomes and susceptibility to illness. However, abnormally strengthened cell death may promote the generation of conditions related to retrogradation. All genes correlated with natural immunoreaction, multiplication, necrocytosis, and differentiation are regulated via ROS production [26]. Apoptosis dysregulation is an obvious pathology in PCOS progression, and the levels of apoptotic markers have been investigated in PCOS [27]. Recently, multiple signaling pathways, including FoxO3, Akt-mTOR, and BMP15-Smad1, have been shown to be involved in regulating GC death in PCOS 28, 29, 30. However, whether the JAK1/STAT3 pathway is related to GC death in PCOS has not been reported. Wang et al. suggested that LEP expression in Th1 cells of PCOS subjects has a positive relationship with subsequent cell death [31]. The present study showed that silencing of LEP-induced apoptosis of GCs treated with insulin, accompanied by activation of the JAK1/STAT3 pathway. The use of curcumin, which could deactivate STAT3 by blocking its phosphorylation and nuclear translocation, counteracted the stimulatory effect of LEP silencing on the death of GCs. LEP may, therefore, play a role in the apoptosis of GCs in PCOS through the JAK1/STAT3 pathway.

In summary, in this study, the authors attempted to explore the function and underlying mechanism of action of LEP in a cell PCOS model, which is established by treating GCs with insulin. The results demonstrated that upregulation of LEP level in the PCOS GC model is essential for reducing apoptosis and inflammation via regulating JAK1/STAT3 pathway, while deactivation of JAK1/STAT3 cascade partially counteracted the function of LEP on inflammation and apoptosis of insulin-treated GCs. Therefore, LEP can be further developed as a prognostic biomarker for PCOS.

## Funding

This research did not receive any specific grant from funding agencies in the public, commercial, or not-for-profit sectors.

## Study guideline

Animal and Clinical Studies should follow the ARRIVE guidelines.

## CRediT authorship contribution statement

**Xuan Zhao:** Conceptualization, Data curation, Formal analysis, Methodology. **Yaming Xiong:** Conceptualization, Data curation, Formal analysis, Methodology. **Ya Shen:** Conceptualization, Data curation, Formal analysis, Validation, Writing – original draft, Writing – review & editing.

## Declaration of competing interests

The authors declare that they have no known competing financial interests or personal relationships that could have appeared to influence the work reported in this paper.
